# IMPACT OF AGING POPULATIONS ON MUNICIPAL EMERGENCY MEDICAL SERVICES

**DOI:** 10.1093/geroni/igz038.3468

**Published:** 2019-11-08

**Authors:** Nidya Velasco Roldan, Caitlin E Coyle, Michael Ward, Jan Mutchler

**Affiliations:** 1 University of Massachusetts Boston, Massachusetts, United States; 2 Gerontology Institute, University of Massachusetts Boston, Boston, Massachusetts, United States; 3 University of Massachusetts Boston, Boston, Massachusetts, United States

## Abstract

The services that residents require from their local governments vary depending on the demographics of their populations. While municipalities have long sought to consider how changes in the young population may impact their school system needs, few systematic considerations have been developed relating to how aging populations may impact municipal service provision. This study aims to address this issue by focusing on demands on emergency services at the municipal level. Using data from the Massachusetts Ambulance Trip Record Information System (MATRIS) we explore the association between emergency medical services (EMS) demand and population age-structure. The data shows an overrepresentation of older people among EMS users. People age 65 and older represent 16% of Massachusetts’ population but account for 31% of the transported emergent calls —e.g., 911 calls— and 60% of the scheduled transports. Results from the OLS regression analysis suggest that communities with larger shares of older residents have significantly higher numbers of EMS calls. The type of community and other age-related community features such as the percentage of older residents living alone and the percentage of older population dually eligible for Medicare and Medicaid are also significantly associated with the number of EMS calls. Contrary to our expectations, other resources available in the community such nursing homes or assisted living facilities were not significantly associated with number of EMS calls. Our research indicates that if growth in the older population occurs as projected, the demand placed on the EMS system by older populations will grow considerably in coming decades.

